# Automation and Microfluidics for the Efficient, Fast, and Focused Reaction Development of Asymmetric Hydrogenation Catalysis

**DOI:** 10.1002/cssc.202200333

**Published:** 2022-06-03

**Authors:** Robbert van Putten, Natalie S. Eyke, Lorenz M. Baumgartner, Victor L. Schultz, Georgy A. Filonenko, Klavs F. Jensen, Evgeny A. Pidko

**Affiliations:** ^1^ Inorganic Systems Engineering Department of Chemical Engineering Delft University of Technology Van der Maasweg 9 2629HZ Delft Netherlands; ^2^ Department of Chemical Engineering Massachusetts Institute of Technology 77 Massachusetts Avenue 02139 Cambridge Massachusetts United States

**Keywords:** asymmetric catalysis, automation, homogeneous catalysis, hydrogenation, microfluidics

## Abstract

Automation and microfluidic tools potentially enable efficient, fast, and focused reaction development of complex chemistries, while minimizing resource‐ and material consumption. The introduction of automation‐assisted workflows will contribute to the more sustainable development and scale‐up of new and improved catalytic technologies. Herein, the application of automation and microfluidics to the development of a complex asymmetric hydrogenation reaction is described. Screening and optimization experiments were performed using an automated microfluidic platform, which enabled a drastic reduction in the material consumption compared to conventional laboratory practices. A suitable catalytic system was identified from a library of Ru^II^‐diamino precatalysts. In situ precatalyst activation was studied with ^1^H/^31^P nuclear magnetic resonance (NMR), and the reaction was scaled up to multigram quantities in a batch autoclave. These reactions were monitored using an automated liquid‐phase sampling system. Ultimately, in less than a week of total experimental time, multigram quantities of the target enantiopure alcohol product were provided by this automation‐assisted approach.

## Graphical abstract

**Faster and more efficient by automation**: Application of automation and microfluidic tools to the development of a homogeneously‐catalyzed asymmetric hydrogenation of a pharmaceutically‐relevant substrate is described herein. In less than one week, multigram quantities of the target γ‐aminoalcohol were produced by the automation‐assisted workflow, using a fraction of the resources required for a typical reaction development campaign.

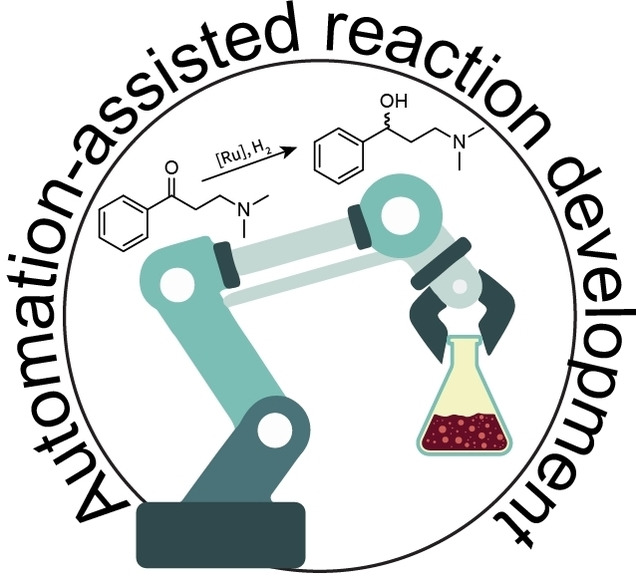

## Introduction

Homogeneous catalysis is a powerful tool that enables complex chemical transformations to be performed efficiently in both industry and academia. The potential economic‐ and ecological savings upon its implementation are significant, even though reaction development of homogeneously catalyzed reactions is often challenging. Such complications arise from the vast parameter space that governs catalytic performance, as well as the relatively unpredictable reactivity of many catalytic systems. These complications can make rational navigation of the available parameter space difficult, tedious, and time consuming. (The well‐documented chiral switch of (*S*)‐Metolachlor took 14 years!^[1]^) In these situations it is often more attractive to screen and model (a section of) the available parameter space using high‐throughput experimentation (HTE) and Design of Experiment (DoE) methods instead.^[2]^

Although these techniques are highly effective, there are also several disadvantages to using HTE.[^2a^, ^2b^, ^2c^, ^2d^] For example, material consumption can be rather high (e. g., substrate, reagents, consumables). Operational limitations of the equipment can also severely limit experimental design. Most parallel screening equipment offers only limited (if any) control over temperature and pressure for different reaction zones. Multiple plates are therefore required to screen different reaction conditions. Because of this relative inflexibility, parallel HTE approaches cannot practically cover the available parameter space at high resolution. This limitation can lead to ‘missing‐out’ of more favorable catalytic systems under conditions that were not experimentally evaluated. Unfortunately, such concerns are not merely theoretical: our recent study on catalytic ketone reduction with a Mn‐based homogeneous catalyst revealed a situation where a 10 °C reduction of the reaction temperature led to a more than five‐fold increase of the catalyst's lifetime (TON turnover number).^[3]^

The ability to simultaneously screen and optimize discrete catalytic parameters (e. g., precatalysts) under individually‐optimized conditions (e. g., continuous reaction parameters such as concentration, pressure, temperature) could address this limitation and drive adoption in the field of homogeneous catalysis. Automated microfluidic tools potentially offer an elegant solution to this problem.^[4]^

Jensen and co‐workers have previously developed an automated microfluidic platform that combined good experimental throughput and flexibility with high‐quality chromatographic analysis.^[5]^ The introduction of a mixed‐integer non‐linear optimization algorithm improved the system‘s ability to optimize reaction conditions in non‐trivial parameter spaces, as are often encountered with catalytic multiphase reactions. This capability has recently been demonstrated with catalytic applications in Suzuki‐Miyaura cross‐couplings^[5d]^ and Buchwald‐Hartwig aminations.^[5i]^

**Figure 1:**
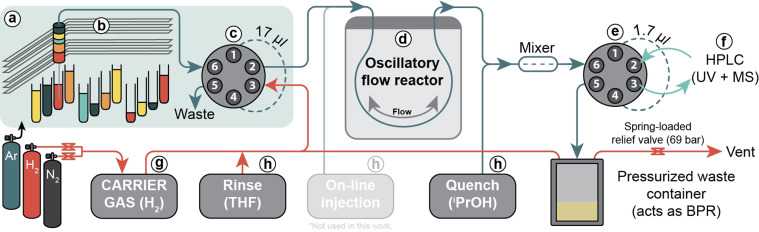
Schematic overview of the automated microfluidic platform used in this work. (a) Argon‐filled glove bag. (b) Gilson GX‐241 liquid handler with stock solutions. (c) Six‐port two‐position injection valve. (d) Oscillatory flow reactor. (e) Six‐port two‐position sampling valve. (f) On‐line Agilent 1260 Infinity HPLC+MS. (g) Carrier gas syringe pump. (h) Rinse, injection, and quench syringe pumps. (On‐line injection was not used in this work; BPR=back pressure regulator).

The oscillatory microfluidic flow platform consists of a liquid handling robot placed inside an Ar‐filled glove bag, four syringe pumps, a custom‐made aluminum reactor block, and an on‐line high performance liquid chromatography (HPLC) system (Figure 1). Reactions are performed inside FEP (Fluorinated Ethylene Propylene) or PFA (Perfluoroalkoxy Alkane) polymer tubing. Discrete reaction mixtures (e. g., to screen different precatalysts) are automatically prepared by the liquid handling robot according to a randomized (computer‐generated) experimental design. A single 17 μL droplet of the reaction mixture is subsequently injected into the gas‐filled system and moved around by the carrier gas. The droplet is oscillated inside the temperature‐controlled reactor for the target residence time. Afterwards, the mixture is quenched and/or diluted, and analyzed with on‐line HPLC. Key performance indicators such as reagent conversion and product yield are computed, and the optimization algorithm is updated with the new information. This cycle is repeated until an optimal solution is found.

The droplet's oscillatory movement is critical to reach the desired Taylor flow pattern, which is characterized by a strong internal circulation that continuously ‘refreshes’ the gas‐liquid interface.^[6]^ The oscillating motion of the liquid inside the microreactor decouples its mixing with the gas‐phase from its residence time and enables experiments with vastly divergent residence times to be performed in the same reactor.^[5b]^ Furthermore, the small scale of these experiments (<1.0 mg substrate, around 0.1 mg metal complex, around 100 μL solvent) ensures low material consumption. These characteristics therefore make oscillatory microfluidics a promising technique to study high‐pressure, gas‐liquid (catalytic) chemistries.^[7]^

Here, we describe our efforts to develop an automated‐assisted workflow for rapid reaction development of high‐pressure gas‐liquid (catalytic) chemistry. This approach was demonstrated with the Ru‐catalyzed asymmetric hydrogenation of a sensitive β‐aminoketone. An automated microfluidic optimization platform was used to conveniently screen discrete precatalysts and identify favorable reaction conditions. The reaction was subsequently scaled up to multigram quantities without significant modifications to the procedure, and reaction progress was monitored with an automated sampling device. This automation‐assisted workflow ultimately afforded chemically‐ and optically pure (*S*)‐**2** in less than a week of total experimental time.

**Scheme 1:**
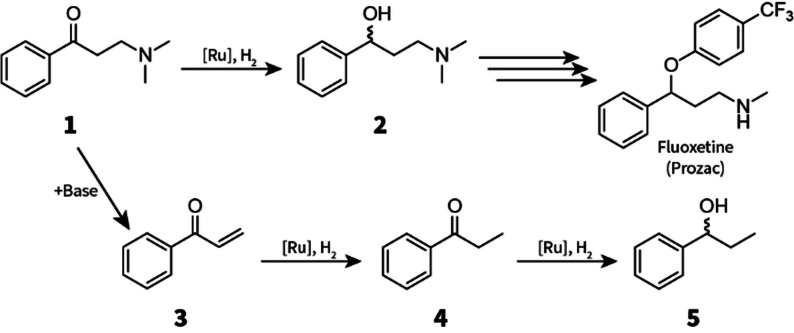
Asymmetric hydrogenation of β‐aminoketone **1** to chiral γ‐aminoalcohol **2** en route Fluoxetine. The side‐reaction of Mannich base **1** with bases leads to (further reducible) acrylophenone (**3**), propiophenone (**4**), and 1‐phenyl‐1‐propanol (**5**).^[8]^

We decided to demonstrate the automated, high‐pressure microfluidic platform with a challenging asymmetric hydrogenation reaction. We selected the chiral reduction of β‐aminoketone **1** as a representative model reaction that could reasonably be encountered in a typical reaction development campaign in the pharmaceutical or fine‐chemical industry (Scheme 1).^[8]^ Asymmetric hydrogenations are commonly applied in these industries for the atom‐efficient (late‐stage) installation of chiral centers. Additionally, the resulting chiral γ‐aminoalcohol is an important building block for the synthesis of several antidepressants and is a potential intermediate en route enantiopure Fluoxetine (which is marketed under the name of Prozac by Eli Lilly and Co.).^[9]^

The asymmetric hydrogenation of **1** met a number of criteria that made it a suitable demonstration reaction. First of all, substrate **1** is largely incompatible with transition metal ketone hydrogenation catalysts. Most precatalysts for these reactions (specifically Ru^II^ chlorides) require the addition of a strong base activator. The base converts the precatalyst into a deprotonated complex that can form catalytically‐active Ru‐hydrides under H_2_ pressure. This requirement for basic conditions is problematic for Mannich bases such as **1**, because exposure leads to undesirable elimination reactions and degradation of the substrate, thereby causing poor yields and/or product selectivity.[^8^, ^10c^, ^10d^]

**Scheme 2:**
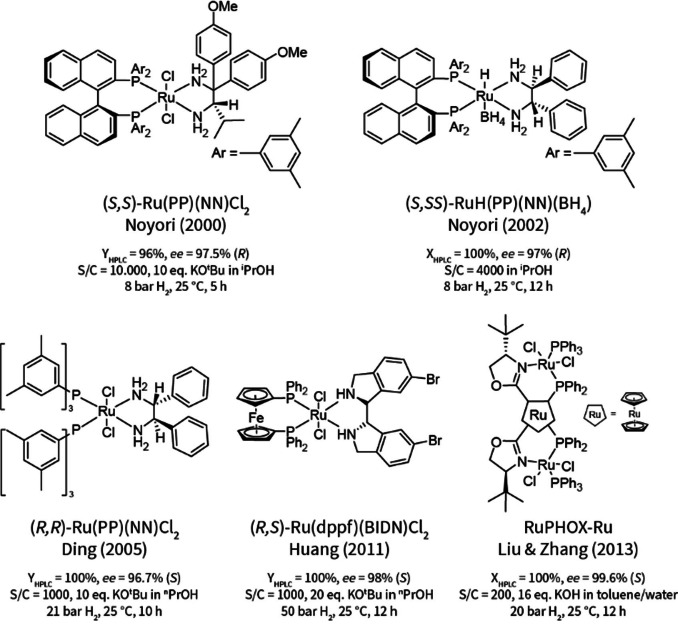
Selected literature examples of the Ru‐catalyzed asymmetric hydrogenation of β‐aminoketone **1** to γ‐aminoalcohol **2**.[^10c^, ^10d^, ^10e^, ^10h^, ^10i^]

Despite this, there are some reports on the catalytic (asymmetric) hydrogenation of **1**.^[10]^ These examples include the use of Rh^I^ or Ru^II^ precatalysts, which resulted in comparable yield and optical purity of **2**. Two strategies have been devised that work around the base sensitivity of **1**: i) catalyst activation with a minimum amount of base to limit its impact, and ii) reaction with a pre‐activated, isolated Ru‐borohydride adduct. The best performing catalytic systems reported so far are summarized in Scheme 2 (full details in Figure S3, Supporting Information). Under optimized conditions all catalytic systems provided **2** in excellent yield at very high enantioselectivity.

The availability of some literature precedent represented an advantage of the selected chemistry for the development and the demonstration of the automated platform. Sufficient information was available to focus our campaign, while it was sufficiently sparse to justify the need for additional screening and optimization. Previous studies pointed to chiral Ru^II^‐diamine as the most potent precatalysts. Furthermore, the relatively high catalyst loadings and reaction times of the reported chemistry also gave room for further optimization. In applied research, such a scenario could, for example, occur if restrictions on the use of intellectual property are encountered and specific compounds cannot be used.

## Results and Discussion

### Automated catalyst screening and optimization

**Scheme 3:**
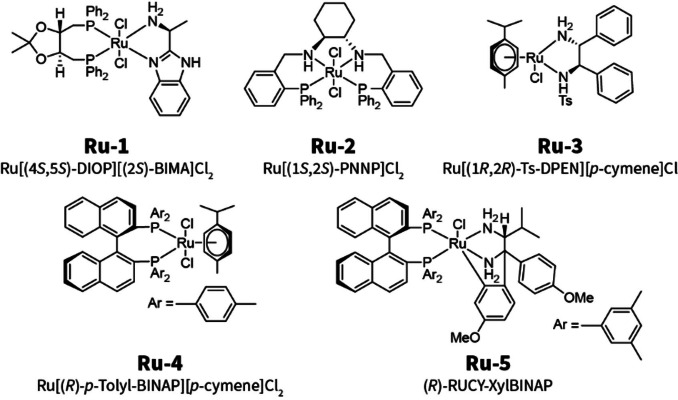
Selection of Ru^II^‐diamine precatalysts used in this work.

We assembled a modest library of structurally‐related complexes that were both commercially available and that had not been used before for the target reaction (Scheme 3). These precatalysts were activated in situ with the addition of five equivalents of NaHBEt_3_ (Sodium triethylborohydride) (see Supporting Information). Addition of the hydride reagent to a solution of the precatalyst in tetrahydrofuran (THF) at room temperature resulted in a pronounced and rapid colour change from yellow/green to golden orange. This in situ preactivation protocol allowed us to efficiently and conveniently screen a series of Ru^II^ precatalysts without the additional workload of (pre)catalyst isolation.

**Figure 2:**
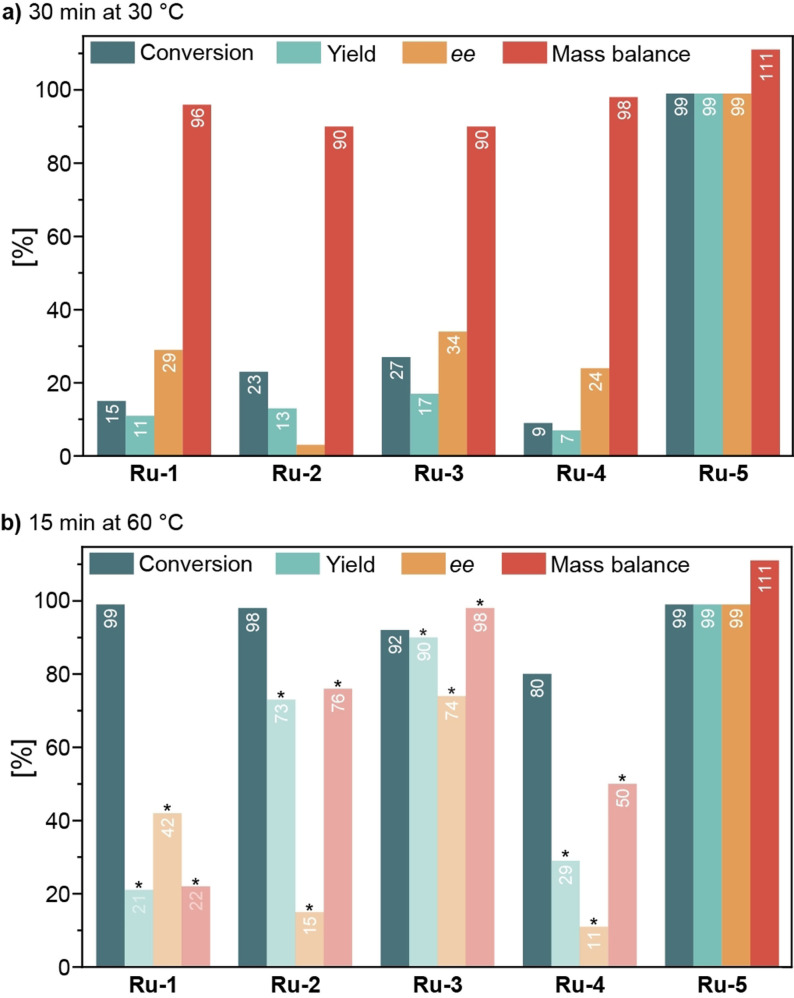
Asymmetric hydrogenation of β‐aminoketone **1** to γ‐aminoalcohol **2** with activated precatalysts **Ru‐1** to **Ru‐5**. Conditions: 0.1 M **1** in 2‐propanol, 3 mol% activated Ru, 15–30 min, 30–60 °C, 30 bar H_2_. Yields were determined by HPLC using 1‐fluoronaphthalene as an internal standard. Translucent data: unresolved side‐product present in chromatogram. Peak integration is unreliable. Data are presented for the sake of completeness.

The reaction development campaign was started with a user‐defined pre‐screening to scout the experimental parameter space. We estimated that a catalyst loading of 3 mol% would allow the evaluation of catalytic performance in approximately 30 min per experiment. Two sets of conditions were evaluated for each catalytic system: i) a ‘long’ experiment of 30 min at 30 °C, and ii) a ‘short’ experiment of 15 min at 60 °C. The results of these experiments are summarized in Figure 2 and Table S1.

At 30 °C, catalysts **Ru‐1** to **Ru‐4** exhibited similar activity and selectivity. ‘RUCY’‐catalyst **Ru‐5** performed significantly better and provided **2** in excellent yield and enantiomeric excess. The experiments at a higher temperature (60 °C) revealed a diverging behavior of the catalysts. Reactions with **Ru‐1** to **Ru‐4** at 60 °C showed a much‐improved conversion of the starting material, and, at first glance, improved product enantiomeric excess. However, close inspection of chromatograms revealed that a chromatographically‐unresolved side‐product was present that had been automatically integrated with the target product. This side‐product was identified as (*RS*)‐1‐phenyl‐1‐propanol (**5**), which was not formed in the lower‐temperature reaction at 30 °C. This compound overlapped with (*S*)‐**2** in the chromatogram, and the automated integration algorithm failed to differentiate between the two overlapping peaks, resulting in an artificially increased apparent yield and *ee* (Enantiomeric excess) of the target product. The expected acrylophenone **3** and propiophenone **4** side‐products were not detected by the HPLC analysis. Side‐product **5** was not observed after reaction with **Ru‐5** at 60 °C; at full conversion, this catalyst produced practically identical reaction mixtures under both the low‐ and high‐temperature conditions.

The obtained experimental results provided several important insights into the (catalytic) chemistry under study. First, the rate of the retro‐Mannich side‐reaction was negligible at 30 °C and was comparable to the rate of the target hydrogenation at 60 °C in the presence of **Ru‐1** to **Ru‐4**. This is in line with the anticipated higher activation energy of the side‐reaction compared to the target catalytic conversion. Secondly, because **3** and **4** were not detected in these experiments, we concluded that they were more readily reduced than substrate **1**. In view of the extraordinary hydrogenation activity observed with **Ru‐5**, it was selected for further studies and the scale‐up reaction. (As suitable conditions were immediately identified, the automated optimization algorithm was not used.)

These results also underlined that automation is a very powerful tool that enables data‐rich, yet resource‐efficient experimentation. When applied strategically, it is more accurate, reproducible, and can be operated (semi‐) continuously to shorten development time with ≥10x reduced material consumption. However, as this case demonstrates, it is not a proverbial ‘silver bullet’. The automated system must still be provided with a robust chromatographic method and its actions should be closely monitored by the operator.

### Precatalyst activation

**Figure 3:**
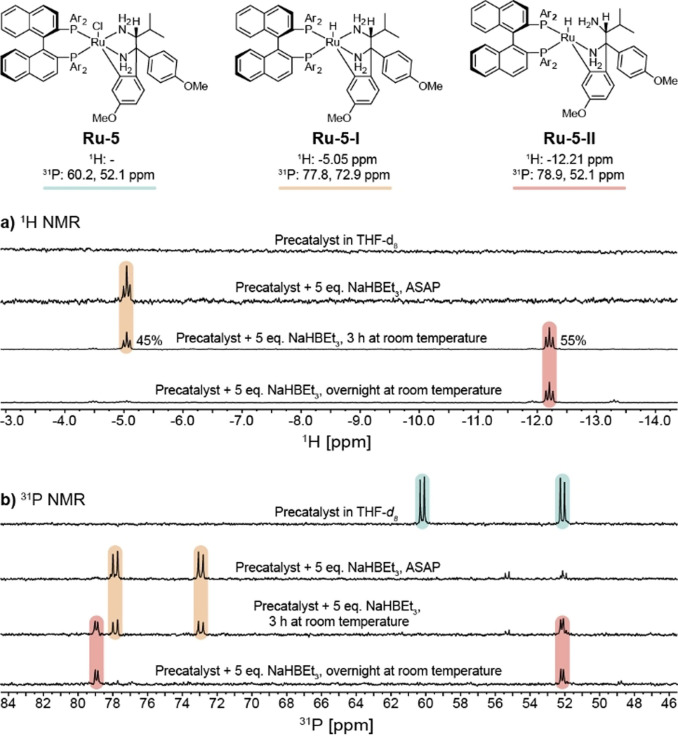
NMR study into activation of (*R*)‐RUCY‐XylBINAP **Ru‐5** with 5 equiv. NaHBEt_3_ in THF‐*d_8_* at room temperature. Approximate reaction times are indicated in the figure. Assignment of Ru‐hydrides **Ru‐5‐I** and **Ru‐5‐II** are consistent with those from Matsumura et al.^[11]^ (a) Time series of ^1^H NMR spectra (400 MHz). (b) Time series of ^31^P NMR spectra (162 MHz).

The in situ catalyst activation process was studied in more detail to support optimization of the targeted reaction scale‐up. The transformation of precatalyst **Ru‐5** upon activation was monitored with ^1^H and ^31^P nuclear magnetic resonance (NMR) spectroscopy. The reaction of (*R*)‐RUCY‐XylBINAP **Ru‐5** with 5 equiv. NaHBEt_3_ in THF‐*d_8_* resulted in a rapid and pronounced color change from green to golden orange. Spectra were acquired immediately after mixing, after approximately 3 h at room temperature, and after a night at room temperature (Figure 3).

Treatment of the Ru^II^ chloride precursor **Ru‐5** with NaHBEt_3_ gave rise to two new Ru‐hydride species. Directly after mixing the solution contained **Ru‐5‐I** as the major species. This complex was characterized by the hydride resonance in the ^1^H NMR spectrum at δ=−5.05 ppm. The phosphorus nuclei of this complex appeared in the ^31^P NMR spectrum as two doublets at 77.8 ppm and 72.9 ppm (^2^ *J*_PP_=43.6 Hz). After 3 h at room temperature the spectrum had changed significantly and a second Ru‐hydride species **Ru‐5‐II** was observed, which was present in a comparable amount with **Ru‐5‐I** (45 % **Ru‐5‐I**, 55 % **Ru‐5‐II)**. The Ru−H resonance of **Ru‐5‐II** appeared significantly upfield at δ=−12.21 ppm compared to that in **Ru‐5‐I**. One of the two doublets in the ^31^P NMR spectrum was also shifted upfield (78.9 ppm and 52.1 ppm with ^2^ *J*_PP_=26.6 Hz). After one night at room temperature the solution contained **Ru‐5‐II** as the major species.

The spectra of complexes **Ru‐5‐I** and **Ru‐5‐II** are consistent with reports from Matsumura et al.,^[11]^ who activated **Ru‐5** with 1 atm H_2_ in the presence of KO^t^Bu (Potassium tert‐butoxide) and observed the two species with multidimensional NMR experiments. **Ru‐5‐I** was identified as an octahedral Ru‐hydride complex that was highly active for the (asymmetric) hydrogenation of ketones. Penta‐ligated complex **Ru‐5‐II** was considerably less catalytically active, which was attributed to the decoordination of the (potentially reactive) amino group. We therefore concluded that the activated precatalyst solution was best used as soon as possible after mixing.

### Reaction scale‐up

**Figure 4:**
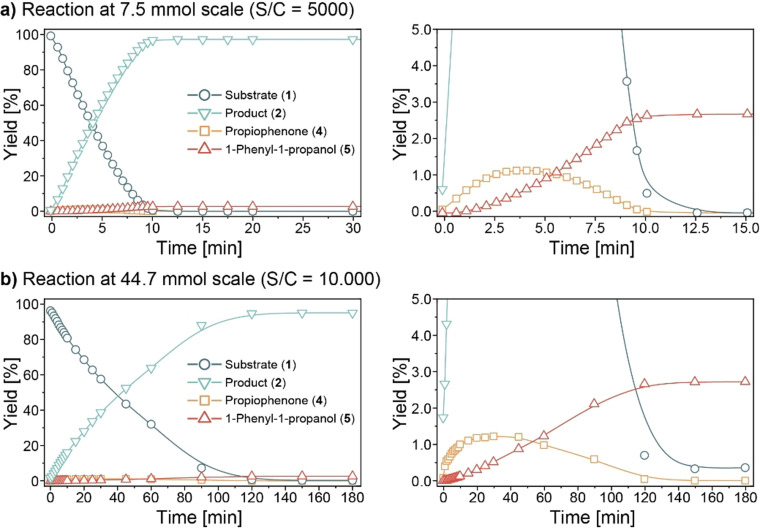
Liquid phase concentration profiles of the asymmetric hydrogenation of **1** to **2** with pre‐activated **Ru‐5**. Samples were withdrawn with a custom‐built automated sampling system. Yields were determined by GC‐FID using *n*‐dodecane as an internal standard. Product enantiomeric excess was determined by UPLC and was constant at >99 % (representative chromatogram in Figure S8). Note that the presence of gas‐liquid mass transfer limitations was not explicitly evaluated for these experiments. (a) Conditions: 7.5 mmol **1** in 20 mL 2‐propanol, 0.02 mol% activated **Ru‐5** (S/C=5,000), 29 °C, 30 bar H_2_. (b) Conditions: 44.7 mmol **1** in 20 mL 2‐propanol, 0.01 mol% activated **Ru‐5** (S/C=10,000), 29 °C, 30 bar H_2_.

The scaled‐up asymmetric hydrogenation reaction was performed twice at different reaction scale and catalyst loading. Reactions were performed in a stainless‐steel autoclave that was fitted with a custom‐built automatic sampling system (Figure 4).^[12]^ This Autosampler system was designed for automated high‐resolution kinetic measurements and enabled us to accurately monitor (very fast) reaction progress and selectivity without the difficulties and cost (labor, consumables) involved of conventional (manual) kinetic experimentation.

The reaction with 0.02 mol% Ru (S/C=5,000, substrate to catalyst ratio) was very fast and completed within 15 min at 29 °C (Figure 4a). Product (*S*)‐**2** was formed in 97 % yield. Product *ee* (not shown) was constant throughout the reaction at >99 % (*S*). Our analysis also allowed the detection of the minor propiophenone (**4**) and 1‐phenyl‐1‐propanol (**5**) products, which were present at only 1–3 % throughout the reaction. Acrylophenone **3** presumably was too reactive and could not be observed by GC‐FID (Gas chromatography with flame ionization detector) or UPLC (Ultra high performance liquid chromatography) analysis. (Note that the reaction mixture was sampled at excessive temporal resolution to demonstrate the Autosampler's capabilities. This was not required for the chemistry under study and diminished the isolated yield of (*S*)‐**2**. It does, however, show the potential of automated data‐rich experimentation for accurate tracking of fast reactions and dilute/transient impurity profiling.)

The reaction's final scale‐up was performed at 0.01 mol% **Ru‐5** (S/C=10,000) with 44.7 mmol (around 8 g) **1** (liquid phase concentration profiles in Figure 4b). This reaction was considerably slower than that at S/C=5,000 and completed in approximately 2.5 h. (*S*)‐**2** was obtained in 97 % GC‐yield at >99 % enantiomeric excess. The fully hydrogenated side‐product **5** was formed in approximately 3 % yield. The target product was isolated from the reaction mixture as its hydrochloride salt. Additional purification by recrystallization from ethanol/diethyl ether ultimately provided 5.81 g of the chemically‐ and enantiomerically pure (*S*)‐**2**⋅HCl product (84 % isolated yield based on residual reaction mixture, 65 % isolated yield on basis of initial substrate loading. Approximately 19 % of the material was removed during sampling).

Our automation‐assisted workflow successfully identified a suitable catalytic system (precatalyst and reaction conditions) from a set of previously untested, commercially available precatalysts. The final catalytic system outperformed the state‐of‐the‐art^[7]^ and provided the target γ‐aminoalcohol product in comparable yield and catalyst loading, at approximately halved reaction time, and improved product enantiopurity (>99 % versus 97.5 %). Importantly, all development was achieved in under a week of total experimental time, thereby highlighting the potential of automation to intensify product development and significantly reduce time‐to‐market.

## Conclusion

Automation and microfluidic tools offer efficient, fast, and focused reaction development of complex chemistries such as homogeneously‐catalyzed asymmetric hydrogenations. Development and implementation of such tools is therefore expected to drive adoption of atom‐ and/or energy‐ efficient catalytic technologies in academic‐ and industrial‐ process research.

Herein, we described the application of automation and microfluidics to the development of a complex, catalytic asymmetric hydrogenation reaction of a sensitive β‐aminoketone substrate. Screening and optimization experiments were performed using an automated microfluidic platform. Favorable reaction conditions were identified from a set of discrete Ru^II^‐diamino precatalysts. In situ precatalyst activation was studied with dedicated nuclear magnetic resonance (NMR) experiments and the reaction was scaled‐up in a batch autoclave reactor system. These reactions were monitored using automated, high‐resolution kinetic analysis using a custom‐built Autosampler system. Ultimately, our automation‐assisted approach produced multigram quantities of the target enantiopure γ‐aminoalcohol product in less than a week of total experimental time using a fraction of the resources required for a conventional reaction development campaign.

## Experimental Section

### Screening and optimization experiments

Screening and optimization experiments were performed using a modified version of the automated microfluidic oscillatory flow platform described in previous works.[^5a^, ^5c^, ^5d^, ^5e^, ^5f^, ^5g^, ^5h^, ^5i^, ^5j^] Modifications were made to the system to enable sustained operation at elevated temperature and pressure (see Supporting Information for details).

### In situ precatalyst activation for NMR study

Inside an Ar‐filled glovebox, 4.0 mg (1.0 equiv., 3.38 μmol) (*R*)‐RUCY‐XylBINAP was dissolved in 0.7 mL THF‐*d_8_* inside an air‐free NMR tube, and ^1^H and ^31^P spectra were measured as soon as possible. 16.9 μL NaHBEt_3_ in THF (1 M, 5.0 equiv., 16.9 μmol) was added, the mixture was shaken, and spectra were acquired immediately, after approximately 3 h, and the next morning. Spectra and assignments are consistent with those reported in literature.^[11]^

### Large scale hydrogenation of 1

Reactions were performed in a 60 mL stainless steel Parr autoclave, equipped with a Julabo CF30 thermostat, and a gas burette system that enabled monitoring of gas consumption. Samples were periodically removed from the system with a custom‐made automated sampling (Autosampler) system.^[12]^ Before the experiment, the autoclave was evacuated at 80 °C for ≥1 h, cooled to 30 °C, and refilled with Ar.

The following is a representative procedure for both experiments: inside the glove box, a glass vial was loaded with 250 μL *n*‐dodecane, 20.0 mL 2‐propanol, and 7.92 g 1 (44.7 mmol). A separate vial was loaded with 1.490 mL of the pre‐activated catalyst solution (4.47 μmol Ru, S/C=10,000). The two mixtures were transferred to the autoclave without exposure to air. The substrate solution was added to the main reactor vessel, and the catalyst solution was placed inside a separate stainless‐steel compartment that was fluidically decoupled from the reactor with a ball valve. Stirring was engaged at 700 rpm and the reaction mixture was preheated for 5 min until it reached the desired temperature (T_Set_=30 °C, T_Internal_=29 °C, measured with an internal thermocouple). Hydrogen pressure was applied (30 bar) and the reaction was started following the addition of the catalyst solution to the reaction mixture at *t*=0 s. Samples were periodically removed from the reaction mixture and were analyzed as described. Afterwards the reactor was depressurized, purged with Ar, and the crude reaction mixture was worked‐up as described below.

### Isolation of (*S*)‐2⋅HCl

After the asymmetric hydrogenation was complete, the crude reaction mixture was dried over MgSO_4_, filtered, and 22.5 mL of a 4.0 M HCl solution in 1,4‐dioxane (90.0 mmol, 2.0 equiv.) was slowly added. The mixture was stirred for approximately 10 min and volatiles were removed in vacuo to yield the crude product as a yellow oil. The oil was triturated for 1 h with 15 mL anhydrous THF to give the product as a white solid. The solids were collected, dried, and recrystallized from ethanol/diethyl ether at −20 °C to give the analytically pure title compound as white crystals. Yield: 5.81 g (84 % isolated yield based on residual reaction mixture, 65 % isolated yield on basis of initial substrate loading. Approximately 19 % of the material was removed during sampling.)

1

## Supporting information

As a service to our authors and readers, this journal provides supporting information supplied by the authors. Such materials are peer reviewed and may be re‐organized for online delivery, but are not copy‐edited or typeset. Technical support issues arising from supporting information (other than missing files) should be addressed to the authors.

Supporting Information

## Data Availability

The data that support the findings of this study are available in the supplementary material of this article.

## References

[1] H. U. Blaser , Adv. Synth. Catal. 2002, 344, 17–31.

[2a] S. M. Mennen , Org. Process Res. Dev. 2019, 23, 1213–1242;

[2b] M. Shevlin , ACS Med. Chem. Lett. 2017, 8, 601–607; 28626518 10.1021/acsmedchemlett.7b00165 PMC5467193

[2c] S. W. Krska , D. A. DiRocco , S. D. Dreher , M. Shevlin , Acc. Chem. Res. 2017, 50, 2976–2985; 29172435 10.1021/acs.accounts.7b00428

[2d] R. Grainger , S. Whibley , Org. Process Res. Dev. 2021, 25, 354–364;

[2e] B. Cao , L. A. Adutwum , A. O. Oliynyk , E. J. Luber , B. C. Olsen , A. Mar , J. M. Buriak , ACS Nano 2018, 12, 7434–7444; 30027732 10.1021/acsnano.8b04726

[2f] J. A. Jurica , J. P. McMullen , Org. Process Res. Dev. 2021, 25, 282–291.

[3] R. van Putten , J. Benschop , V. J. de Munck , M. Weber , C. Müller , G. A. Filonenko , E. A. Pidko , ChemCatChem 2019, 11, 5232–5235. 31894188 10.1002/cctc.201900882 PMC6919935

[4a] A. R. Bogdan , A. W. Dombrowski , J. Med. Chem. 2019, 62, 6422–6468; 30794752 10.1021/acs.jmedchem.8b01760

[4b] K. F. Jensen , AIChE 2017, 63, 858–869;

[4c] M. B. Plutschack , B. Pieber , K. Gilmore , P. H. Seeberger , Chem. Rev. 2017, 117, 11796–11893. 28570059 10.1021/acs.chemrev.7b00183

[5a] M. Abolhasani , C. W. Coley , L. Xie , O. Chen , M. G. Bawendi , K. F. Jensen , Chem. Mater. 2015, 27, 6131–6138;

[5b] M. Abolhasani , N. C. Bruno , K. F. Jensen , Chem. Commun. 2015, 51, 8916–8919; 10.1039/c5cc02051d 25876959

[5c] B. J. Reizman , K. F. Jensen , Chem. Commun. 2015, 51, 13290–13293; 10.1039/c5cc03651h 26201048

[5d] B. J. Reizman , Y. M. Wang , S. L. Buchwald , K. F. Jensen , React. Chem. Eng. 2016, 1, 658–666; 27928513 10.1039/c6re00153j PMC5123644

[5e] C. W. Coley , M. Abolhasani , H. Lin , K. F. Jensen , Angew. Chem. Int. Ed. 2017, 56, 9847–9850; 10.1002/anie.201705148 28651035

[5f] Y. J. Hwang , C. W. Coley , M. Abolhasani , A. L. Marzinzik , G. Koch , C. Spanka , H. Lehmann , K. F. Jensen , Chem. Commun. 2017, 53, 6649–6652; 10.1039/c7cc03584e 28585652

[5g] L. M. Baumgartner , C. W. Coley , B. J. Reizman , K. W. Gao , K. F. Jensen , React. Chem. Eng. 2018, 3, 301–311;

[5h] H. W. Hsieh , C. W. Coley , L. M. Baumgartner , K. F. Jensen , R. I. Robinson , Org. Process Res. Dev. 2018, 22, 542–550;

[5i] L. M. Baumgartner , J. M. Dennis , N. A. White , S. L. Buchwald , K. F. Jensen , Org. Process Res. Dev. 2019, 23, 1594–1601;

[5j] M. Abolhasani , K. F. Jensen , Lab Chip 2016, 16, 2775–2784. 27397146 10.1039/c6lc00728g

[6a] P. Angeli , A. Gavriilidis , in Encyclopedia of Microfluidics and Nanofluidics (Ed.: D. Li ), Springer US, Boston, MA, 2008, pp. 1971–1976;

[6b] M. T. Kreutzer, Ph.D. thesis, Delft University of Technology (Netherlands), **2003**;

[6c] P. Bianchi , J. D. Williams , C. O. Kappe , J. Flow Chem. 2020, 10, 475–490.

[7] C. Zhu , K. Raghuvanshi , C. W. Coley , D. Mason , J. Rodgers , M. E. Janka , M. Abolhasani , Chem. Commun. 2018, 54, 8567–8570. 10.1039/c8cc04650f 29989636

[8a] T. Tőrös , L. Kollàr , B. Heil , J. Organomet. Chem. 1982, 232, C17–C18;

[8b] T. Kosmalski , Acta Pol. Pharm. 2010, 67, 717–721; 21229895

[8c] M. Tramonti , L. Angiolini , in Mannich bases, CRC press, 1994, p. 83.

[9a] D. T. Wong , J. S. Horng , F. P. Bymaster , K. L. Hauser , B. B. Molloy , Life Sci. 1974, 15, 471–479; 4549929 10.1016/0024-3205(74)90345-2

[9b] B. B. Molloy, K. K. Schmiegel, US4018895 A, **1977**;

[9c] B. B. Molloy, K. K. Schmiegel, US4584404 A, **1983**;

[9d] C. J. Wenthur , M. R. Bennett , C. W. Lindsley , ACS Chem. Neurosci. 2014, 5, 14–23.

[10a] H. Takahashi , S. Sakuraba , H. Takeda , K. Achiwa , J. Am. Chem. Soc. 1990, 112, 5876–5878;

[10b] M. Devocelle , F. Agbossou , A. Mortreux , Synlett 1997, 1997, 1306–1308;

[10c] T. Ohkuma , D. Ishii , H. Takeno , R. Noyori , J. Am. Chem. Soc. 2000, 122, 6510–6511;

[10d] T. Ohkuma , M. Koizumi , K. Muñiz , G. Hilt , C. Kabuto , R. Noyori , J. Am. Chem. Soc. 2002, 124, 6508–6509; 12047151 10.1021/ja026136+

[10e] Q. Jing , X. Zhang , J. Sun , K. Ding , Adv. Synth. Catal. 2005, 347, 1193–1197;

[10f] H. L. Ngo , W. Lin , J. Org. Chem. 2005, 70, 1177–1187; 15704949 10.1021/jo048333s

[10g] P. D. de Koning , M. Jackson , I. C. Lennon , Org. Process Res. Dev. 2006, 10, 1054–1058;

[10h] Q. Zhu , D. Shi , C. Xia , H. Huang , Chem. Eur. J. 2011, 17, 7760–7763; 21618636 10.1002/chem.201100820

[10i] J. Wang , D. Liu , Y. Liu , W. Zhang , Org. Biomol. Chem. 2013, 11, 3855–3861; 23657215 10.1039/c3ob40135a

[10j] W. Xu , R. Langer , Dalton Trans. 2015, 44, 16785–16790. 26339700 10.1039/c5dt02226f

[11] K. Matsumura , N. Arai , K. Hori , T. Saito , N. Sayo , T. Ohkuma , J. Am. Chem. Soc. 2011, 133, 10696–10699. 21675799 10.1021/ja202296w

[12a] R. van Putten, E. A. Uslamin, E. A. Pidko, WO2021162552, **2020**;

[12b] R. van Putten , E. A. Uslamin , E. A. Pidko , Invention Disclosure 2021, 1, 100002.

