# Supplementary material for: Automation and Microfluidics for the Efficient, Fast, and Focused Reaction Development of Asymmetric Hydrogenation Catalysis
Source: ChemSusChem. 2022 Jun 3;15(14):e202200333. doi: 10.1002/cssc.202200333 (PMC9401021; doi:10.1002/cssc.202200333)
Supplement: Supplementary file 1 — Supporting Information [file CSSC-15-0-s001.pdf]

ChemSusChem

Supporting Information

Automation and Microfluidics for the Efficient, Fast, and Focused Reaction Development of Asymmetric Hydrogenation Catalysis

Robbert van Putten, Natalie S. Eyke, Lorenz M. Baumgartner, Victor L. Schultz, Georgy A. Filonenko, Klavs F. Jensen,* and Evgeny A. Pidko*© 2022 The Authors. ChemSusChem published by Wiley-VCH GmbH. This is an open access article under the terms of the Creative Commons Attribution License, which permits use, distribution and reproduction in any medium, provided the original work is properly cited.

SUPPORTING INFORMATION

Automation and microfluidics for the efficient, fast, and focused reaction development of asymmetric hydrogenation catalysis

Robbert van Putten[a,b], Natalie S. Eyke[b], Lorenz M. Baumgartner[b], Victor L. Schultz[b], Georgy A. Filonenko[a], Klavs F. Jensen[b]*, Evgeny A. Pidko[a]*

[a] Inorganic Systems Engineering, Department of Chemical Engineering, Faculty of Applied Sciences, Delft University of Technology, Van der Maasweg 9, 2629 HZ, Delft, The Netherlands

[b] Department of Chemical Engineering, Massachusetts Institute of Technology, 77 Massachusetts Avenue, Cambridge, Massachusetts 02139, United States

Corresponding authors:

Klavs F. Jensen (kfjensen@mit.edu) & Evgeny A Pidko (E.A.Pidko@tudelft.nl)

Contents

S1 – General Considerations S3
S2 – Synthetic Procedures S4
1 - 3-(Dimethylamino)propriophenone S4
2 - (*S*)-3-(Dimethylamino)-1-phenyl-1-propanol hydrochloride S5
S3 – Screening and optimization experiments S6
System construction S6
Precatalyst activation S8
Automated screening and optimization experiments – procedure S9
Automated screening and optimization experiments – literature overview S11
Automated screening and optimization experiments – results S12
S4 – Reactivity study with (*R*)-RUCY-XylBINAP S15
S5 – Large scale hydrogenations S16
Activation of (*R*)-RUCY-XylBINAP for large scale hydrogenations S16
Large scale hydrogenation of **1** S16
S6 – Analytical details S21
Analytical details – GC-FID S21
Analytical details – HPLC/UPLC S21
S7 – References S22

S1 – General Considerations

All manipulations were, unless stated otherwise, performed under an inert atmosphere in an Ar-filled Inert glove box or using standard Schlenk techniques. Anhydrous solvents were dispensed from an Inert PureSolv solvent purification system or were dried using 3 or 4 Å molecular sieves. Solvents were degassed before use. Chemicals were purchased from Sigma-Aldrich, Strem, TCI, or Key Organics and were dried and/or degassed before use. Air and/or moisture sensitive materials were stored in the glove box. Deuterated solvents were purchased from Eurisotop, dried using molecular sieves, degassed, and stored in the glove box. Complex **Ru-2** was prepared according to literature procedure.[1-2]

NMR spectra were recorded on an Agilent 400-MR DD2 400 MHz spectrometer equipped with a 5 mm ONE NMR probe. ^{1}H and ^{13}C chemical shifts were referenced to residual solvent peaks (^{1}H: 3.58 ppm THF-d_8, 7.26 ppm $CDCl_3$. ^{13}C: 67.21 ppm THF-d_8, 77.16 ppm $CDCl_3$). ^{31}P NMR spectra were referenced via the IUPAC absolute chemical shift (Ξ = 40.480742).

S2 – Synthetic Procedures

1 - 3-(Dimethylamino)propriophenone

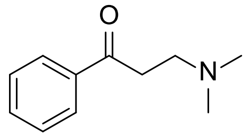

1

Compound **1** was freebased from the commercially available hydrochloride salt via the procedure below, or was prepared *via* a Mannich reaction following an adapted procedure from Almutairi *et al.*[3]

To a 500 ml flask was added 24.0 g acetophenone (200 mmol, 1.0 eq.), 22.0 g dimethylamine hydrochloride (270 mmol, 1.35 eq.), 8.1 g paraformaldehyde (270 mmol, 1.35 eq.), 1.0 ml concentrated aqueous HCl, and 200 ml anhydrous ethanol. The mixture was refluxed overnight in air.

Afterwards volatiles were removed *in vacuo*, the resulting solids were filtered off, washed with cold acetone, and dried under vacuum to give 35.6 g of the crude product as a white solid. At this stage the main contamination was residual dimethylamine hydrochloride.

The hydrochloride salts were dissolved in 200 ml water and were cooled to 0 °C. To the solution was added dropwise a 3.65 M solution of KOH in water at 0 °C until the pH reached >12. The product was extracted with diethyl ether (4 x 50 ml). Combined organic phases were dried over $MgSO_4$ and the solvent was removed *in vacuo* to obtain the product as a clear liquid that was further purified by crystallization. The liquid was cooled to -20 °C to induce crystallization. The large colorless crystals were filtered off, briefly dried under vacuum, and transferred to the glove box for storage. The title compound was obtained as a colorless, slightly viscous liquid. Yield: 23.7 g (67%). Purity: 99.3% (GC relative area%).

^{1}H NMR (400 MHz, $CDCl_3$): δ 7.94–7.88 (m, aryl *H*, 2H), 7.53–7.47 (m, aryl *H*, 1H), 7.44–7.36 (m, aryl *H*, 2H), 3.09 (t, *J* = 7.3 Hz, CH_2, 2H), 2.70 (d, *J* = 7.9 Hz, CH_2, 2H), 2.23 (s, $N(CH_3)_2$, 6H).

^{13}C NMR (101 MHz, $CDCl_3$): all resonances are singlets: δ 199.0, 136.9, 133.0, 128.6, 128.0, 54.4, 45.5, 36.9.

2 - (*S*)-3-(Dimethylamino)-1-phenyl-1-propanol hydrochloride

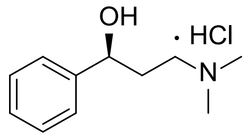

(*S*)-2·HCl

After the asymmetric hydrogenation was complete (see procedure in **Section S5**), the crude reaction mixture was dried over $MgSO_4$, filtered, and 22.5 ml of a 4.0 M HCl solution in 1,4-dioxane (90.0 mmol, 2.0 eq.) was slowly added. The mixture was stirred for approximately 10 minutes and volatiles were removed *in vacuo* to yield the crude product as a yellow oil. The oil was triturated for 1 h with 15 ml anhydrous THF to give the product as a white solid. The solids were collected, dried, and recrystallized from ethanol/diethyl ether at -20 °C to give the analytically pure title compound as white crystals. Yield: 5.81 g (65% of initial loading, 84% of residual reaction mixture after sample withdrawal. 19% of the material was removed during sampling).

^{1}H NMR (400 MHz, D_2O): δ 7.55–7.36 (m, aryl *H*, 5H), 4.90–4.82 (m, 1H), 3.36–3.22 (m, 1H), 3.22–3.09 (m, 1H), 2.89 (s, N(CH_3)$_2$, 6H), 2.30–2.11 (m, CH_2, 2H).

^{13}C NMR (101 MHz, D_2O): all resonances are singlets: δ 142.5, 129.0, 128.4, 125.9, 71.3, 55.3, 43.0, 42.7, 32.4.

S3 – Screening and optimization experiments

System construction

Screening and optimization experiments were performed using a modified version of the automated microfluidic oscillatory flow platform that was described in previous works (**Figure S1**).[4-12]. Modifications were made to the system to enable sustained operation at elevated temperature and pressure (see below). Microfluidic tubing and connectors were purchased from IDEX Health and Services (system: FEP 1/16" OD x 0.02" ID, reactor: PFA 1/8" OD x 1/16" ID).

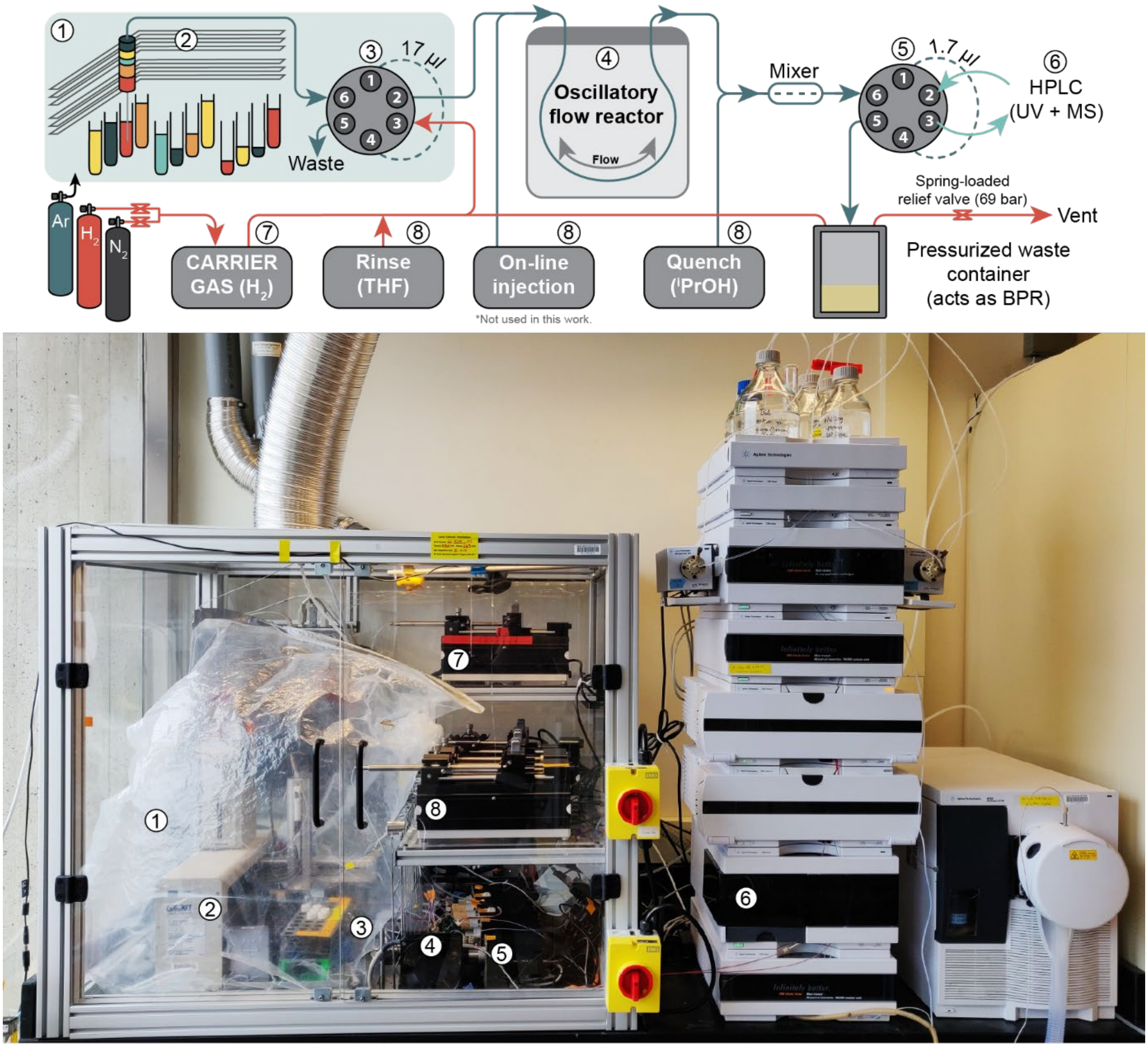

① Ar-filled glove bag
② Gilson GX-241 liquid handler with stock solutions
③ Six-port two-position injection valve (not visible in picture)
④ Oscillatory flow reactor (not visible in picture)
⑤ Six-port two-position sampling valve
⑥ On-line Agilent 1260 Infinity HPLC + MS
⑦ Carrier gas syringe pump
⑧ Rinse, injection, and quench syringe pumps

Figure S1. Overview of the automated microfluidic oscillatory flow platform used in this work.

A number of system modifications were required to enable high-pressure operation with hazardous gases. Prolonged experimentation at elevated temperature and pressure (~50 °C at 69 bar) caused the polymer tubing to extrude where it was no longer supported by the Al reactor body (**Figure S2a**). A ¼” (6.35 mm) hole was therefore drilled into the reactor body, and a stainless steel annular ring was press-fit into it to provide back pressure for the tubing (**Figure S2b**). This also physically distanced the unsupported polymer tubing from the heated assembly.

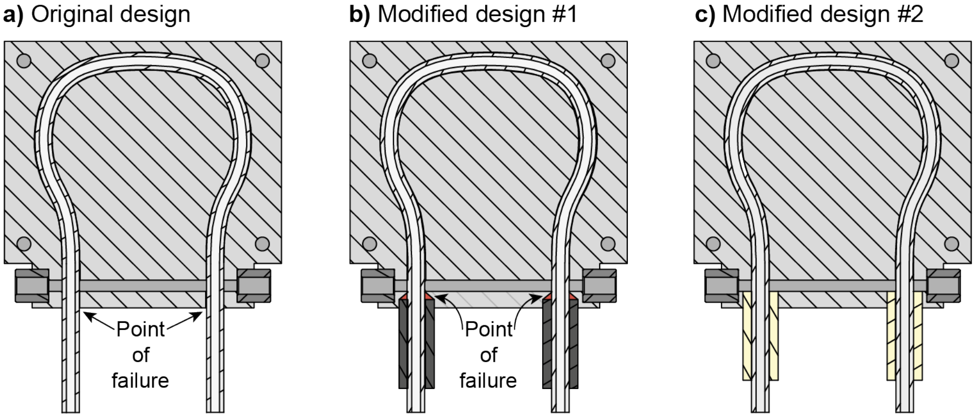

Figure S2. Reactor design iterations. **a)** Original reactor design. **b)** Modified design #1. This design was unsuccessful and failed at the locations indicated in red. **c)** Modified design #2.

This new design had a failure mode that was similar to that of the original design. Extrusion had occurred between the Al reactor body and the stainless steel annular ring (**Figure S2b**). This small area had been left unsupported because a regular (*i.e.*, angled) drill bit had been used for the modification. This situation was corrected with the corresponding end mill. The stainless steel ring was replaced by a PTFE analogue to further reduce thermal conductivity from the Al reactor body to the unsupported sections (**Figure S2c**).

The second design enabled sustained operation at 75 °C and 69 bar for at least three hours. Further heating to 85 °C caused the system to fail after an additional 1.5 hours. This failure occurred in the small gap between the PTFE annular ring and the PEEK fitting that was connected to it (not shown). The observed delay before failure was presumably caused by the relatively non-conductive PTFE ring.

The conditions that could be reached with the new design were adequate for the chiral demonstration chemistry and no further modifications were made. Operational limits were set at 70 °C and 40 bar.

(Higher temperature or pressure in principle are possible, but not simultaneously.) The absolute temperature limit was determined by PFA's glass transition temperature (T_g = 90 °C). The system's maximum operating pressure was determined by the syringe pump's linear force limits. These motors provided approximately 34 kg of linear force. When 8 ml stainless steel syringes were employed, this force limit translated to a maximum pressure of 47 bar. (Use of a smaller 2.5 ml syringe could in principle allow operation at up to 188 bar. High-force syringe pumps are also available from the same supplier.)

Finally, changes were made to the setup and its enclosure to facilitate safe operation. The ventilation rate of the cabinet was improved following the installation of a large-diameter exhaust tube and removal of several 90° bends. The volumetric flow rate was verified to be sufficient to prevent the build-up of hydrogen gas in case of a reasonably-sized leak. A gas detector was installed at the top of the same enclosure to provide early warning of a potential (small) leak. A spring-loaded overpressure relief valve was installed that was connected directly to the exhaust and was set to an opening pressure of 69 bar (1000 psi).

Precatalyst activation

Precatalysts were activated at room temperature inside an N_2-filled glove box by the addition of 5.0 eq. $NaHBEt_3$ (1.0 M in THF) to a solution of the Ru complex in THF. The mixtures were stirred in the glove box for ≥1 h. Immediately upon addition the color of the mixtures changed from yellow/green (depending on the precatalyst) to golden orange.

Note: $NaBH_4$ was initially used for catalyst activation and resulted in similar catalytic activity as $NaHBEt_3$. Use of these suspensions occasionally led to blockages of the microfluidic system, while filtration caused diminished catalytic performance. The procedure based on $NaHBEt_3$ was more convenient and reproducible, and was therefore preferred over activation with $NaBH_4$.

Automated screening and optimization experiments – procedure

Stock solutions of precisely-known concentrations were prepared of the catalysts in 2-propanol, and of substrate **1** in 2-propanol with 1-fluoronaphthalene as an internal standard. The solutions were transferred to conical glass vials, capped with a silicone septum, and taken out of the glove box. The vials were placed inside a vial rack inside the liquid handler module (Gilson GX-241), which itself was placed inside a gas-tight glove bag. 2-Propanol was used as the liquid handler's transfer solvent and the pump was primed. The bag was then purged with Ar until the atmosphere inside measured <0.1 vol% O_2 (measured with a Honeywell GasAlert MicroClip X3 gas detector located inside the bag).

Stainless steel high-pressure syringes (8 ml, Harvard Apparatus) were filled with the appropriate solvent while inside the glove box (THF in rinse, 2-propanol in quench, injection not used), taken outside without exposure to air, placed inside the syringe pumps (Harvard Apparatus PHD Ultra), and were primed. The microfluidic system was thoroughly purged of air through a series of gas-exchange cycles, during which: *i)* the system was pressurized with 30 bar N_2, *ii)* the carrier syringe was fully withdrawn, *iii)* the gas supply was closed, *iv)* the carrier syringe volume was infused into the system, and *v)* the system was depressurized via the waste valve. The cycle was repeated twice more with N_2, after which the procedure was repeated thrice with 30 bar H_2. Experiments were then started and proceeded through phases of sample preparation, reaction, and analysis.

Four 15 µl rinse slugs were injected before each experiment to clean the system and to minimize carry-over between experiments. Reaction mixtures were prepared by the liquid handling robot. A total volume of 40 µl was prepared, which was thoroughly mixed inside the liquid handler, and was injected into a 17 µl sample loop that was connected to a six-port two-position valve inside the glove bag. Once the system was clean and empty, the liquid handler switched the valve and injected the droplet into the H_2-filled system. The slug was then transported to the reactor by slow gas infusion from the carrier syringe. The droplet's location inside the system was tracked with a number of phase sensors (Optek OCB350L062Z).

Once inside the reactor, the droplet was oscillated for the target residence time. The position of the droplet was tracked using a photodetector. This sensor signaled when the droplet was about to exit the reactor and when the carrier flow had to be reversed. Temperature of the custom-made Al reactor block was controlled to ±2.0 °C with cartridge heaters and a fan. After the reaction the droplet was diluted two-fold with 2-propanol, thoroughly mixed, and injected into a 1.7 µl sample loop that was connected to a six-port two-position valve. The sample was injected into the on-line HPLC system and was analyzed as described.

HPLC results were imported into MATLAB, and conversion, yield, and product enantiomeric excess were calculated. The natural logarithm of the absolute value of product enantiomeric excess was used for the optimisation objective function (**Equation S1**).

$$f_{obj} = ln|ee| = ln\left|\frac{Yield_{(R)} - Yield_{(S)}}{Yield_{(R)} + Yield_{(S)}}\right| \qquad \textbf{S1}$$

This was required because *a priori* knowledge on product stereoselectivity for a given catalyst's absolute configuration was not usually available (*i.e.*, the capacity to predict what enantiomer forms preferentially from a given catalyst). Use of absolute enantiomeric excess enabled the maximization of *ee* without the need to assign a target enantiomer. The desired absolute configuration can be accessed with the corresponding catalyst after the screening and optimisation phase. A yield criterion of 0.9 was used to prioritize product *ee* over total yield. No further changes were made to the published optimisation algorithm.[9]

Automated screening and optimization experiments – literature overview

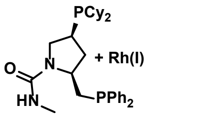

(*S*,*S*)-Rh(I)-MCCPM (1990)
Achiwa[13]
Y_{NMR} = 100 %, *ee* = 63.6 % (*R*)
S/C = 10.000 in MeOH
10 bar H_2, 60 °C, 36 h

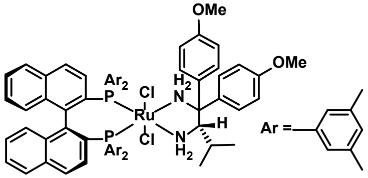

(*S*,*S*)-Ru(diphosphine)(diamine)Cl_2 (2000)
Noyori[14]
Y_{HPLC} = 96 %, *ee* = 97.5 % (*R*)
S/C = 10.000, 10 eq. KO^{t}Bu in ^{i}PrOH
8 bar H_2, 25 °C, 5 h

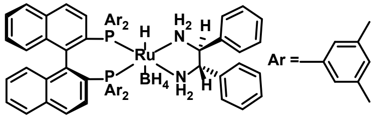

(*S*,*SS*)-RuH(diphosphine)(diamine)(BH_4) (2002)
Noyori[15]
X_{HPLC} = 100 %, *ee* = 97 % (*R*)
S/C = 4000 in ^{i}PrOH
8 bar H_2, 25 °C, 12 h

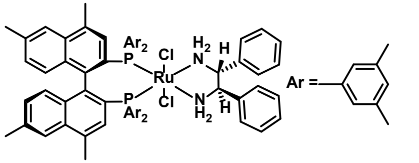

(*R*,*RR*)-RuCl_2(diphosphine)(diamine) (2004)
Lin[16]
***ee* = 98.3 %**
S/C = 1000, 20 eq. KO^{t}Bu in ^{i}PrOH
48 bar H_2, 25 °C, 24 h

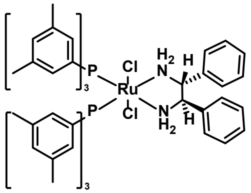

(*R*,*R*)-RuCl_2(phosphine)$_2$(diamine) (2005)
Ding[17]
Y_{HPLC} = 100 %, *ee* = 96.7 % (*S*)
S/C = 1000, 10 eq. KO^{t}Bu in ^{n}PrOH
21 bar H_2, 25 °C, 10 h

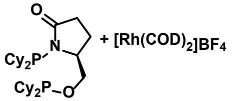

(*S*)-[Rh(COD){AMPP}]BF_4 (1997)
Agbossou[18]
Y_{HPLC} = 90 %, *ee* = 93 % (*R*)
S/C = 200 in MeOH
50 bar H_2, 20 °C, 45 h

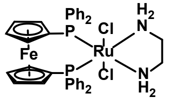

Ru(dppf)(en)Cl_2 (2006)
Jackson[19]
Y = 78 % (racemic HCl salt)
S/C = 5000, 10 eq. KO^{t}Bu in ^{i}PrOH
10 bar H_2, 30 °C, 4 h

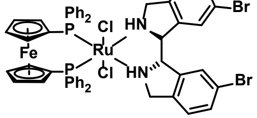

(*R*,*S*)-Ru(dppf)(BIDN)Cl_2 (2011)
Huang[20]
Y_{HPLC} = 100 %, *ee* = 98 % (*S*)
S/C = 1000, 20 eq. KO^{t}Bu in ^{n}PrOH
50 bar H_2, 25 °C, 12 h

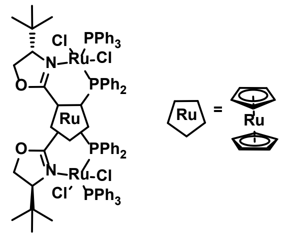

RuPHOX-Ru (2013)
Liu & Zhang[21]
X_{HPLC} = 100 %, *ee* = 99.6 % (*S*)
S/C = 200, 16 eq. KOH in toluene/H_2O
20 bar H_2, 25 °C, 12 h

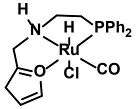

RuHCl(CO)(PNO) (2015)
Langer[22]
Yield not reported
S/C = 200, 200 eq. KOH in H_2O
10 bar H_2, 60 °C, 36 h

Figure S3. Overview of literature reports for the (asymmetric) catalytic reduction of **1** with molecular hydrogen.[13-22]

Automated screening and optimization experiments – results

Experiments were performed as described with a library of structurally-related Ru precatalyst that were both commercially available and had not, to the best of our knowledge, been used before for the target reaction (**Figure S4**). We estimated that a catalyst loading of 3 mol% Ru would allow the evaluation of catalytic performance in approximately 30 minutes per experiment. Experimental results are summarized in **Table S1** and **Figure S5**.

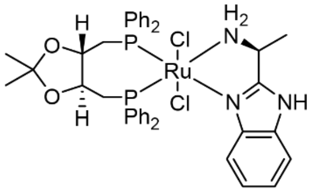

Ru-1
Ru[(*4S,5S*)-DIOP][(*2S*)-BIMA]Cl_2

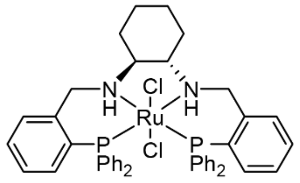

Ru-2
Ru[(*1S,2S*)-PNNP]Cl_2

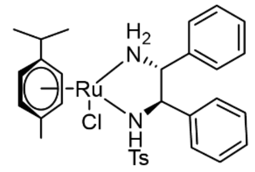

Ru-3
Ru[(*1R,2R*)-Ts-DPEN][*p*-cymene]Cl

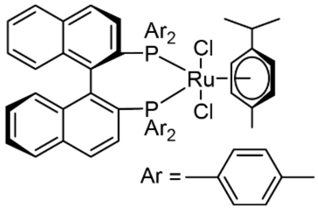

Ru-4
Ru[(*R*)-*p*-Tolyl-BINAP][*p*-cymene]Cl_2

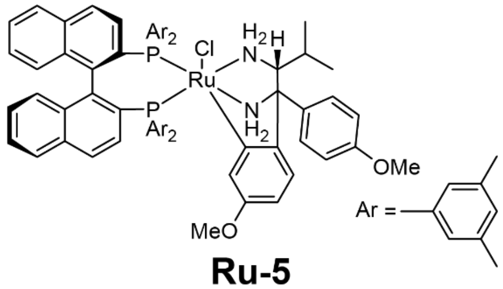

Ru-5
(*R*)-RUCY-XylBINAP

Figure S4. Overview of Ru^{II} precatalysts used in this work for the asymmetric hydrogenation of **1**.

Table S1. Results of screening and optimization experiments.

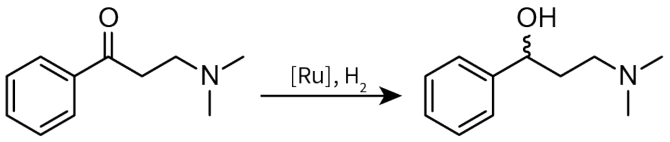

1 **2**

Entry [-]	Time [min]	Temperature [°C]	Precatalyst [-]	Conversion [%]	Yield **2** [%]	*ee* [%]	Mass balance [%]
1[a]			**Ru-1**	99	21	42	22
2[a]			**Ru-2**	98	73	15	76
3[a]	15	60	**Ru-3**	92	90	74	98
4[a]			**Ru-4**	80	29	11	50
5			**Ru-5**	>99	>99	>99	111
6			**Ru-1**	15	11	29	96
7			**Ru-2**	23	13	3	90
8	30	30	**Ru-3**	27	17	34	90
9			**Ru-4**	9	7	24	98
10			**Ru-5**	>99	>99	>99	111

Conditions: 0.1 M **1** in 2-propanol, 3 mol% preactivated Ru, 15-30 min, 30-60 °C, 30 bar H_2. Yields were determined by HPLC using 1-fluoronaphthalene as an internal standard. [a] Unresolved side-product present in chromatogram. Peak integration is unreliable.

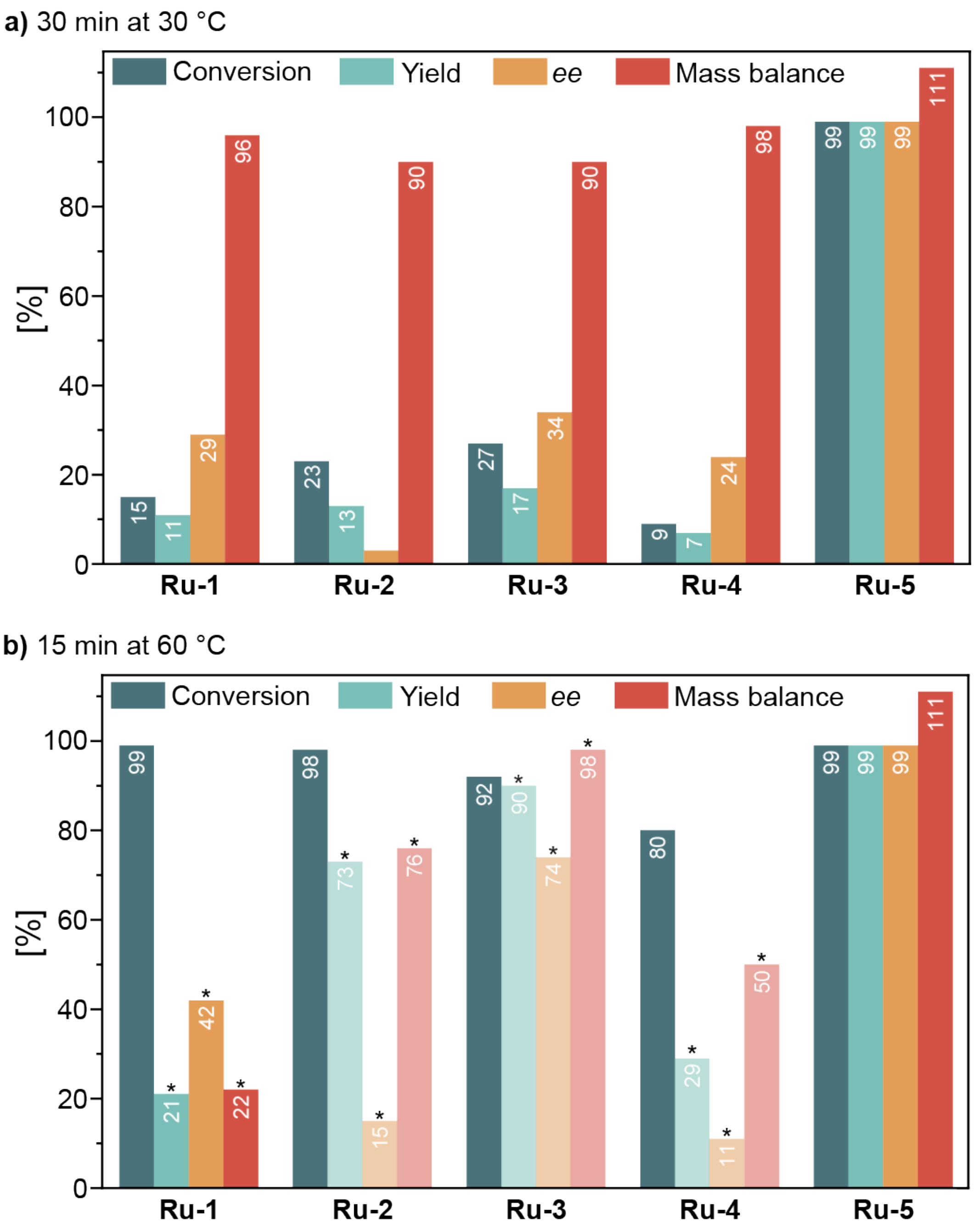

Figure S5. Results of screening and optimization experiments. Translucent data: unresolved side-product present in chromatogram. Peak integration is unreliable. Data are presented for the sake of completeness.

S4 – Reactivity study with (*R*)-RUCY-XylBINAP

In the glovebox, 4.0 mg (1.0 eq., 3.38 μmol) (*R*)-RUCY-XylBINAP was dissolved in 0.7 ml THF-d_8 inside an air-free NMR tube, and ^{1}H and ^{31}P spectra were measured as soon as possible (ASAP). 16.9 μl $NaHBEt_3$ in THF (1 M, 5.0 eq., 16.9 μmol) was added, the mixture was shaken, and spectra were acquired immediately, after approximately 3 hours, and the next morning (**Figure S6**). Spectra and assignments are consistent with those reported by Matsumura *et al.*[23]

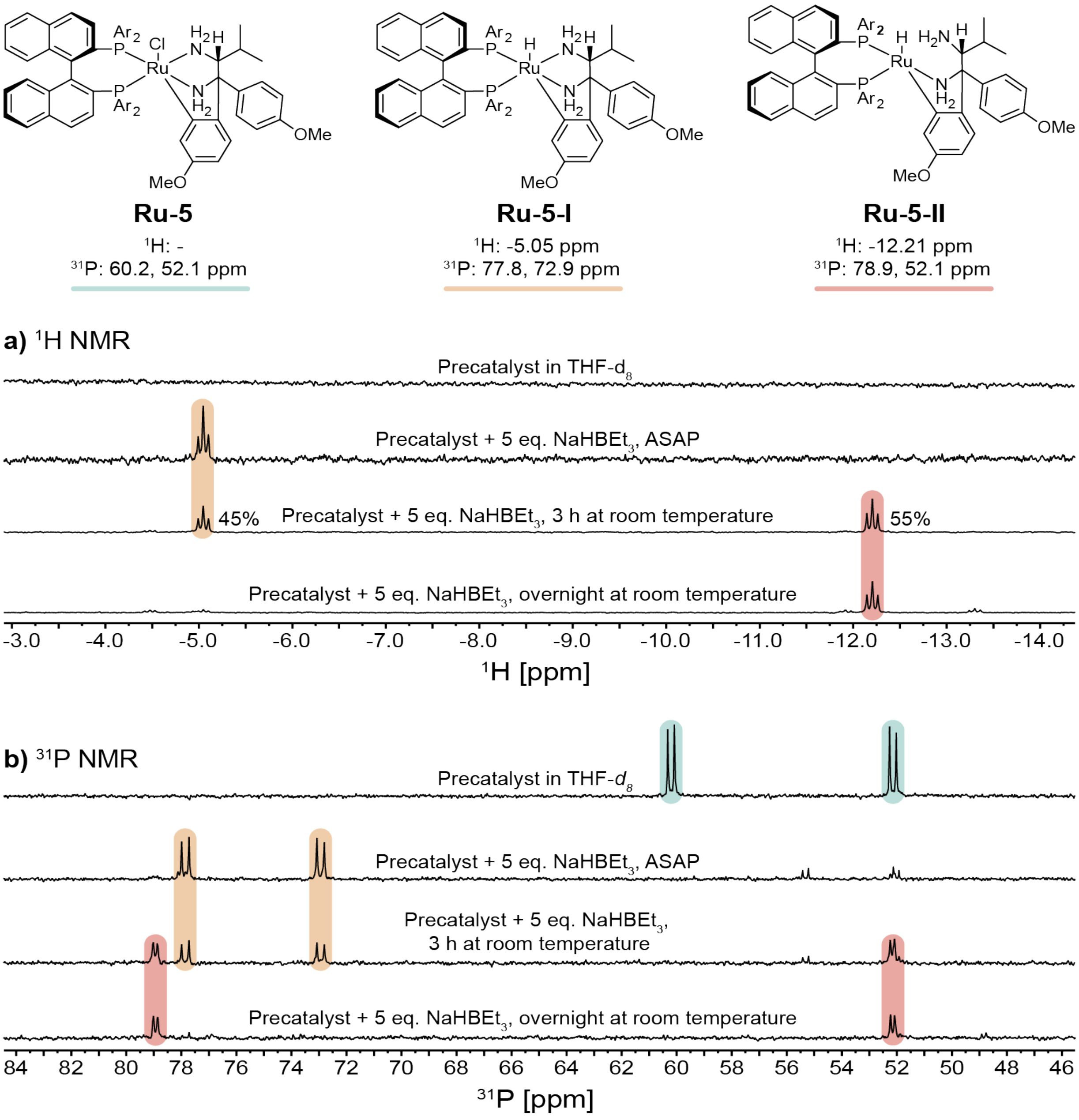

Figure S6. NMR study into activation of (*R*)-RUCY-XylBINAP with 5 eq. $NaHBEt_3$ in THF at room temperature. Approximate reaction times are indicated in the figure. Assignment of Ru hydrides **Ru-5-I** and **Ru-5-II** are from Matsumura *et al.*[23] **a)** ^{1}H NMR spectrum in THF-d_8 (400 MHz). **b)** ^{31}P NMR spectrum in THF-d_8 (162 MHz).

S5 – Large scale hydrogenations

Activation of (*R*)-RUCY-XylBINAP for large scale hydrogenations

Inside the glove box, 14.2 mg (*R*)-RUCY-XylBINAP (12.0 µmol. 1.0 eq.) was dissolved in 4.0 ml THF. To the stirred solution was added 60.0 µl of a 1.0 M $NaHBEt_3$ solution in THF (60.0 µmol, 5.0 eq.). The mixture was stirred at room temperature inside the glove box for ≥1 h. Immediately upon addition, the color of the solution changed rapidly from green/yellow to orange.

Large scale hydrogenation of 1

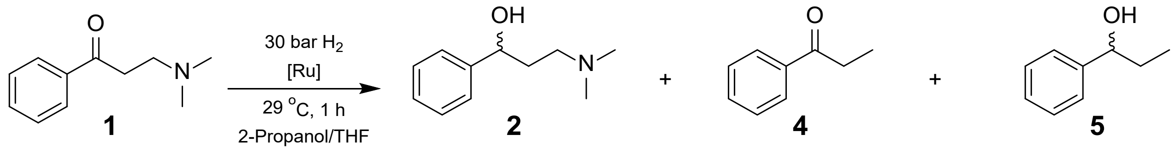

Reactions were performed in a 60 ml stainless steel Parr autoclave, equipped with a Julabo CF30 thermostat and a gas burette system that enabled monitoring of gas consumption. Samples were periodically removed from the system with a custom-made automated sampling system.[24-25] Before the experiment, the autoclave was evacuated at 80 °C for ≥1 h, cooled to 30 °C, and refilled with Ar.

The following is a representative procedure for both experiments: inside the glove box, a glass vial was loaded with 250 µl n-dodecane, 20.0 ml 2-propanol, and 7.92 g 1 (44.7 mmol). A separate vial was loaded with 1.490 ml of the pre-activated catalyst solution (4.47 µmol Ru, S/C = 10.000, see section above). The two mixtures were transferred to the autoclave without exposure to air. The substrate solution was added to the main reactor vessel, and the catalyst solution was placed inside a separate stainless steel compartment that was fluidically decoupled from the reactor with a ball valve.

Stirring was engaged at 700 rpm and the reaction mixture was preheated for 5 minutes until it reached the desired temperature (T_{Set} = 30 °C, $T_{Internal}$ = 29 °C, measured with an internal thermocouple). Hydrogen pressure was applied (30 bar) and the reaction was started following the addition of the catalyst solution to the reaction mixture. Samples were periodically removed from the reaction mixture and were analyzed as described. Afterwards the reactor was depressurized, purged with Ar, and the crude reaction mixture was worked-up as described (**Section S2**). Liquid phase concentration

profiles and final compositions are summarized in **Figure S7** and **Table S2-S3**. A representative chromatogram from chiral UPLC is provided in **Figure S8**.

a) Reaction at 7.5 mmol scale (S/C = 5000)

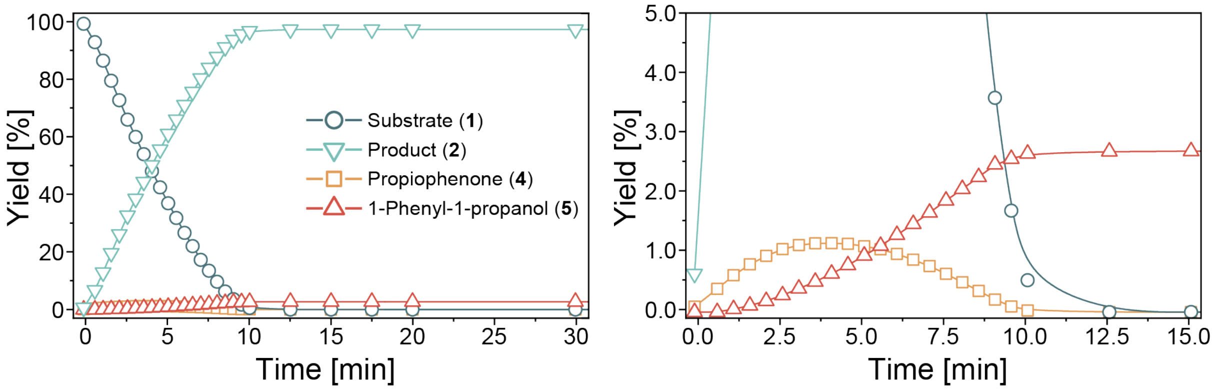

b) Reaction at 44.7 mmol scale (S/C = 10.000)

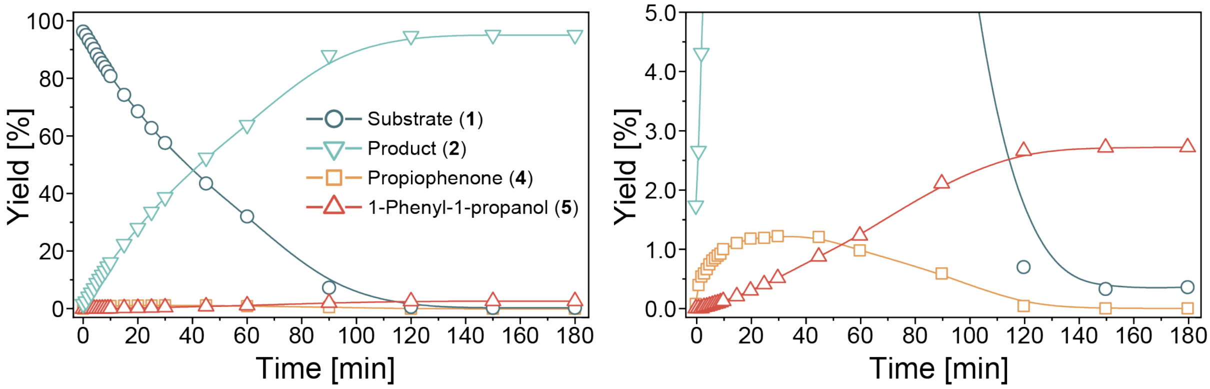

Figure S7. Liquid phase concentration profiles of the asymmetric hydrogenation of **1** to **2** with **Ru-5**. Yields were determined by GC-FID using *n*-dodecane as an internal standard. Product enantiomeric excess was determined by UPLC and was constant at >99% (representative chromatogram in **Figure S8**). Note that the presence of gas-liquid mass transfer limitations was not explicitly evaluated for these experiments. **a)** Conditions: 7.5 mmol **1** in 20 ml 2-propanol, 0.02 mol% activated **Ru-5** (S/C = 5000), 29 °C, 30 bar H_2. **b)** Conditions: 44.7 mmol **1** in 20 ml 2-propanol, 0.01 mol% activated **Ru-5** (S/C = 10.000), 29 °C, 30 bar H_2.

Table S2. Liquid phase concentration profiles for large scale asymmetric hydrogenations of **1** to **2** with **Ru-5**. Yields were determined by GC-FID using n-dodecane as an internal standard. Product enantiomeric excess was determined by UPLC and was constant at >99% (representative chromatogram in **Figure S8**). Conditions: 7.5 mmol **1** in 20 ml 2-propanol, 0.02 mol% activated **Ru-5** (S/C = 5000), 29 °C, 30 bar H_2.

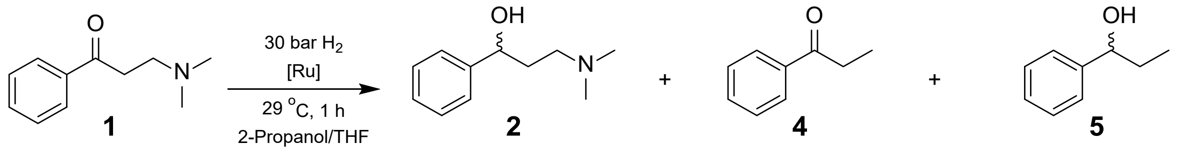

Entry<br>[-]	Time<br>[s]	Time<br>[min]	Conversion<br>[%]	Yield **2**<br>[%]	Yield **4**<br>[%]	Yield **5**<br>[%]
1	0	0	0.7	0.6	0.1	<0.1
2	42	0.7	7.1	6.7	0.4	<0.1
3	72	1.2	13.5	12.9	0.6	<0.1
4	102	1.7	20.5	19.6	0.8	0.1
5	132	2.2	27.3	26.2	0.9	0.2
6	162	2.7	34.0	32.7	1.1	0.3
7	192	3.2	40.0	38.5	1.1	0.4
8	222	3.7	46.0	44.4	1.2	0.5
9	252	4.2	52.0	50.2	1.2	0.6
10	282	4.7	57.6	55.6	1.1	0.8
11	312	5.2	63.1	61.0	1.1	0.9
12	342	5.7	68.2	66.0	1.1	1.1
13	372	6.2	73.4	71.1	1.0	1.3
14	402	6.7	78.0	75.7	0.9	1.5
15	432	7.2	82.7	80.3	0.8	1.7
16	462	7.7	86.5	84.0	0.6	1.9
17	492	8.2	90.4	87.8	0.5	2.1
18	522	8.7	93.8	91.1	0.4	2.3
19	552	9.2	96.4	93.7	0.2	2.5
20	582	9.7	98.3	95.6	0.1	2.6
21	612	10.2	99.5	96.8	<0.1	2.7
22	762	12.7	>99.9	97.3	<0.1	2.7
23	912	15.2	>99.9	97.3	<0.1	2.7
24	1062	17.7	>99.9	97.3	<0.1	2.7
25	1212	20.2	>99.9	97.3	<0.1	2.7
26	1812	30.2	>99.9	97.3	<0.1	2.7
27	2712	45.2	>99.9	97.3	<0.1	2.7
28	3611	60.2	>99.9	97.3	<0.1	2.7

Table S3. Liquid phase concentration profiles for large scale asymmetric hydrogenations of **1** to **2** with **Ru-5**. Yields were determined by GC-FID using n-dodecane as an internal standard. Product enantiomeric excess was determined by UPLC and was constant at >99% (representative chromatogram in **Figure S8**). Conditions: 44.7 mmol **1** in 20 ml 2-propanol, 0.01 mol% activated **Ru-5** (S/C = 10.000), 29 °C, 30 bar H_2.

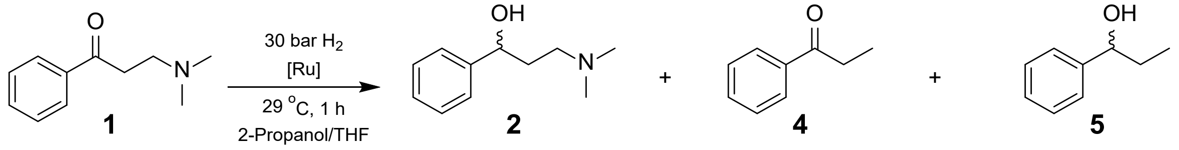

Entry<br>[-]	Time<br>[s]	Time<br>[min]	Conversion<br>[%]	Yield **2**<br>[%]	Yield **4**<br>[%]	Yield **5**<br>[%]
1	2	0.0	1.8	1.7	0.1	<0.1
2	62	1.0	3.1	2.7	0.4	<0.1
3	122	2.0	4.9	4.3	0.5	<0.1
4	182	3.0	6.4	5.8	0.6	<0.1
5	242	4.0	7.9	7.2	0.7	<0.1
6	302	5.0	10.0	9.2	0.7	<0.1
7	362	6.0	11.5	10.7	0.8	0.1
8	422	7.0	13.0	12.1	0.8	0.1
9	482	8.0	14.4	13.4	0.9	0.1
10	542	9.0	16.0	15.0	0.9	0.1
11	602	10.0	17.6	16.5	1.0	0.1
12	902	15.0	24.2	22.9	1.1	0.2
13	1202	20.0	30.1	28.6	1.2	0.3
14	1502	25.0	36.0	34.4	1.2	0.4
15	1802	30.0	41.2	39.5	1.2	0.5
16	2702	45.0	55.6	53.5	1.2	0.9
17	3602	60.0	67.3	65.1	1.0	1.2
18	5402	90.0	92.5	89.8	0.6	2.1
19	7202	120.0	99.3	96.6	<0.1	2.7
20	9002	150.0	99.7	97.0	<0.1	2.7
21	10802	180.0	99.6	96.9	<0.1	2.7

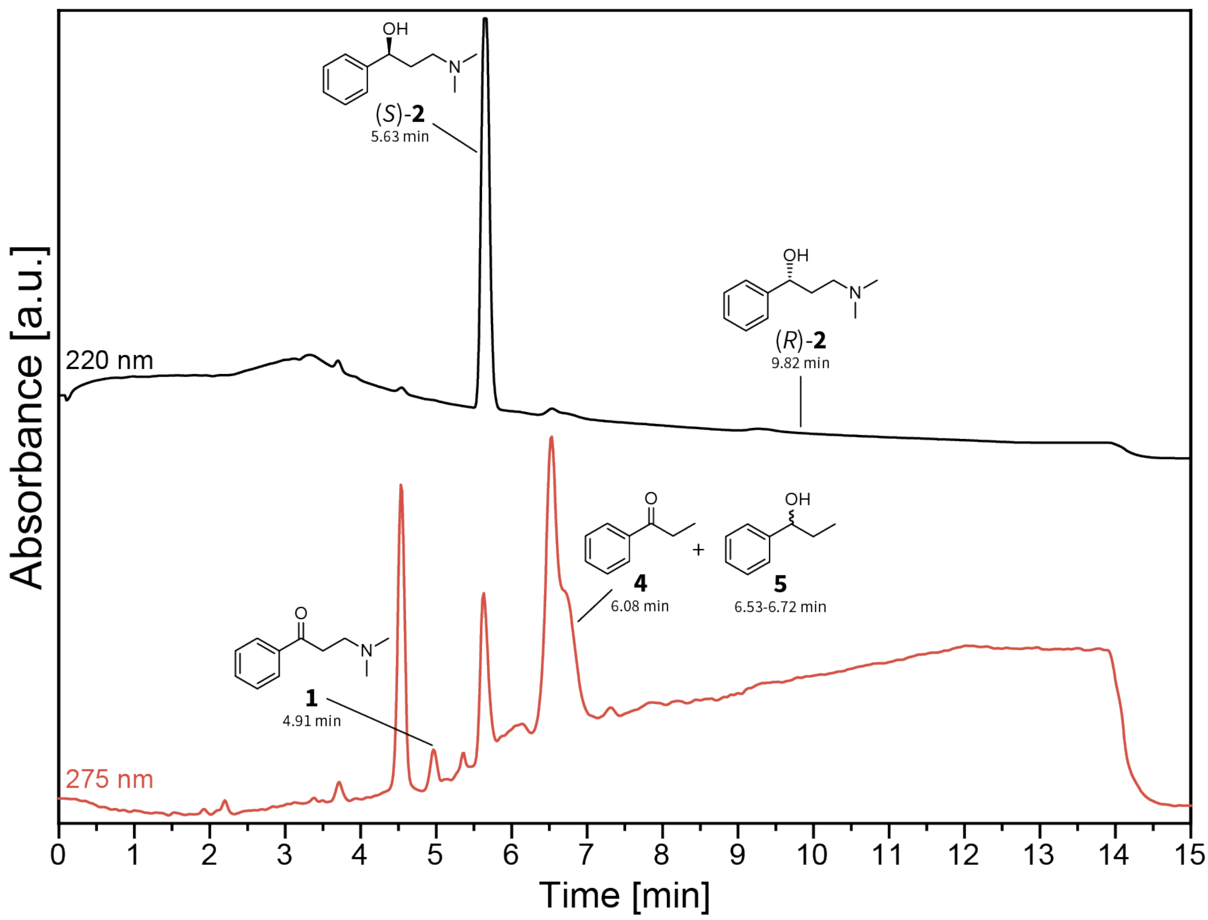

Figure S8. Representative chromatogram at the end of the asymmetric hydrogenation reaction (shown chromatogram was measured after 150 min of 44.7 mmol reaction (**Table S3**, entry 20). Product enantiomeric excess was >99%.

S6 – Analytical details

Analytical details – GC-FID

Measurements were performed on an Agilent 7890B gas chromatograph equipped with FID and Agilent 5977B MS detectors and an Restek Stabilwax-MS column (30 m, 0.32 mmID, 0.25 μm film thickness). Method details: 100 °C (hold 0.5 min), ramp to 250 °C at 30 °C min^{-1} (hold at 250 °C for 3.0 min). Products were identified using the retention times of analytically pure reference samples and by comparison of observed mass spectra and spectra of the authentic reference samples available in the NIST 17 and SDBS libraries. Mass balances were calculated using *n*-dodecane as an internal standard and were, unless stated otherwise, verified to be within 90%–110% for all experiments.

Analytical details – HPLC/UPLC

Measurements were performed on an Agilent 1260 Infinity HPLC or Waters Acquity UPLC. Systems were equipped with UV detectors and were used at 220 nm and 275 nm. Agilent: measurements were performed with a Phenomenex Lux Cellulose-2 column (100 mm, 4.6 μm, 5 mm) at 30 °C. Samples were eluted at 2.0 ml min^{-1} with *n*-heptane/2-propanol/diethylamine (*v*/*v*) as the eluent. Method details: 95/5/0.1 for 0.2 min, ramp to 60/40/0.1 in 4.3 min, hold 0.5 min, ramp to 95/5/0.1 in 0.1 min, hold 0.9 min (total runtime 6.0 min). Waters: measurements were performed with a Phenomenex Lux Cellulose-2 column (250 mm, 4.6 μm, 5 mm) at 30 °C. Samples were eluted at 1.5 ml min^{-1} with *n*-heptane/2-propanol/diethylamine (*v*/*v*) as the eluent. Method details: ramp from 95/5/0.1 to 60/40/0.1 in 10.0 min, hold 2.0 min, ramp to 95/5/0.1 in 0.1 min, hold 2.9 min (total runtime 15.0 min). Products and absolute configuration were identified using the retention times of analytically pure reference samples. Mass balances were calculated using 1-fluoronaphthalene as an internal standard and were, unless stated otherwise, verified to be within 90%–110% for all experiments. (On-line HPLC: mixtures containing >~90% **2** exceeded the linear detector response range for this compound.)

S7 – References

1. Wong, W.K.; Chik, T.W.; Hui, K.N.; Williams, I.; Feng, X.; Mak, T.C.W.; Che, C.M., *Preparation of chiral diimino- and diaminodiphosphine ligands and their CuI and AgI complexes. X-ray crystal structures of [Cu(1S,2S-cyclohexyl-P_2N_2)][PF_6] and [Ag(1R,2R-cyclohexyl-$P_2N_2H_4$)][BF_4]*, *Polyhedron* **1996**, *15* (24), 4447-4460;
2. Wong, W.K.; Chen, X.P.; Pan, W.X.; Guo, J.P.; Wong, W.Y., *Synthesis, Chemistry, and Catalytic Activity of Ruthenium Diaminodiphosphane Complexes − Crystal Structures of trans-[$RuCl_2\{\kappa^3$-$Ph_2PC_6H_4CH{=}NC_6H_{10}N(H)CH_2C_6H_4PPh_2\}(PPh_3)$] and cis-[$RuCl_2\{\kappa^4$-$Ph_2PC_6H_4CH{=}NC_6H_{10}N(H)CH_2C_6H_4PPh_2\}$]*, *Eur. J. Inorg. Chem.* **2002**, *2002* (1), 231-237;
3. Almutairi, M.S.; Jayasheela, K.; Periandy, S.; Al-Ghamdi, A.R.; Sebastian, S.; Xavier, S.; Kadi, A.A.; Abdelhameed, A.S.; Attia, M.I., *Structural, spectroscopic, Hirshfeld surface and charge distribution analysis of 3-(1H-imidazole-1-yl)-1-phenylpropan-1-ol complemented by molecular docking predictions: An integrated experimental and computational approach*, *J. Mol. Struct.* **2019**, *1196*, 578-591;
4. Abolhasani, M.; Coley, C. W.; Xie, L.; Chen, O.; Bawendi, M.G.; Jensen, K.F., *Oscillatory Microprocessor for Growth and in Situ Characterization of Semiconductor Nanocrystals*, *Chem. Mater.* **2015**, *27* (17), 6131-6138;
5. Reizman, B.J.; Jensen, K.F., *Simultaneous solvent screening and reaction optimization in microliter slugs*, *ChemComm* **2015**, *51* (68), 13290-13293;
6. Reizman, B.J.; Wang, Y.M.; Buchwald, S.L.; Jensen, K.F., *Suzuki–Miyaura cross-coupling optimization enabled by automated feedback*, *React. Chem. Eng.* **2016**, *1* (6), 658-666;
7. Coley, C.W.; Abolhasani, M.; Lin, H.; Jensen, K.F., *Material-Efficient Microfluidic Platform for Exploratory Studies of Visible-Light Photoredox Catalysis*, *Angew. Chem. Int. Ed.* **2017**, *56* (33), 9847-9850;
8. Hwang, Y.J.; Coley, C.W.; Abolhasani, M.; Marzinzik, A.L.; Koch, G.; Spanka, C.; Lehmann, H.; Jensen, K.F., *A segmented flow platform for on-demand medicinal chemistry and compound synthesis in oscillating droplets*, *ChemComm* **2017**, *53* (49), 6649-6652;
9. Baumgartner, L.M.; Coley, C.W.; Reizman, B.J.; Gao, K.W.; Jensen, K.F., *Optimum catalyst selection over continuous and discrete process variables with a single droplet microfluidic reaction platform*, *Reaction Chemistry & Engineering* **2018**, *3* (3), 301-311;
10. Hsieh, H.W.; Coley, C.W.; Baumgartner, L.M.; Jensen, K.F.; Robinson, R.I., *Photoredox Iridium–Nickel Dual-Catalyzed Decarboxylative Arylation Cross-Coupling: From Batch to Continuous Flow via Self-Optimizing Segmented Flow Reactor*, *Org. Process Res. Dev.* **2018**, *22* (4), 542-550;
11. Baumgartner, L.M.; Dennis, J.M.; White, N.A.; Buchwald, S.L.; Jensen, K.F., *Use of a Droplet Platform To Optimize Pd-Catalyzed C–N Coupling Reactions Promoted by Organic Bases*, *Org. Process Res. Dev.* **2019**, *23* (8), 1594-1601;
12. Abolhasani, M.; Jensen, K.F., *Oscillatory multiphase flow strategy for chemistry and biology*, *Lab Chip* **2016**, *16* (15), 2775-2784;
13. Takahashi, H.; Sakuraba, S.; Takeda, H.; Achiwa, K., *Asymmetric reactions catalyzed by chiral metal complexes. 41. Highly efficient asymmetric hydrogenation of amino ketone derivatives leading to practical syntheses of (S)-propranolol and related compounds*, *J. Am. Chem. Soc.* **1990**, *112* (15), 5876-5878;
14. Ohkuma, T.; Ishii, D.; Takeno, H.; Noyori, R., *Asymmetric Hydrogenation of Amino Ketones Using Chiral $RuCl_2$(diphophine)(1,2-diamine) Complexes*, *J. Am. Chem. Soc.* **2000**, *122* (27), 6510-6511;

15. Ohkuma, T.; Koizumi, M.; Muñiz, K.; Hilt, G.; Kabuto, C.; Noyori, R., *trans-RuH(η-BH_4)(binap)(1,2-diamine): A Catalyst for Asymmetric Hydrogenation of Simple Ketones under Base-Free Conditions*, *J. Am. Chem. Soc.* **2002**, *124* (23), 6508-6509;
16. Ngo, H.L.; Lin, W., *Development of 4,4'-Substituted-XylBINAP Ligands for Highly Enantioselective Hydrogenation of Ketones*, *J. Org. Chem.* **2005**, *70* (4), 1177-1187;
17. Jing, Q.; Zhang, X.; Sun, J.; Ding, K., *Bulky Achiral Triarylphosphines Mimic BINAP in Ru(II)-Catalyzed Asymmetric Hydrogenation of Ketones*, *Adv. Synth. Catal.* **2005**, *347* (9), 1193-1197;
18. Devocelle, M.; Agbossou, F.; Mortreux, A., *Asymmetric Hydrogenation of α, β, and γ-Aminoketones Catalyzed by Cationic Rhodium(I){AMPP} Complexes*, *Synlett* **1997**, *1997* (11), 1306-1308;
19. de Koning, P.D.; Jackson, M.; Lennon, I.C., *Use of Achiral (Diphosphine)$RuCl_2$(Diamine) Precatalysts as a Practical Alternative to Sodium Borohydride for Ketone Reduction*, *Org. Process Res. Dev.* **2006**, *10* (5), 1054-1058;
20. Zhu, Q.; Shi, D.; Xia, C.; Huang, H., *Ruthenium Catalysts Containing Rigid Chiral Diamines and Achiral Diphosphanes for Highly Enantioselective Hydrogenation of Aromatic Ketones*, *Chem. Eur J.* **2011**, *17* (28), 7760-7763;
21. Wang, J.; Liu, D.; Liu, Y.; Zhang, W., *Asymmetric hydrogenation of β-amino ketones with the bimetallic complex RuPHOX-Ru as the chiral catalyst*, *Org. Biomol. Chem.* **2013**, *11* (23), 3855-3861;
22. Xu, W.; Langer, R., *Probing the effect of heterocycle-bonding in PNX-type ruthenium pre-catalysts for reactions involving H_2*, *Dalton Trans.* **2015**, *44* (38), 16785-16790;
23. Matsumura, K.; Arai, N.; Hori, K.; Saito, T.; Sayo, N.; Ohkuma, T., *Chiral Ruthenabicyclic Complexes: Precatalysts for Rapid, Enantioselective, and Wide-Scope Hydrogenation of Ketones*, *J. Am. Chem. Soc.* **2011**, *133* (28), 10696-10699;
24. van Putten, R.; Uslamin, E.A.; Pidko, E.A., *Sampling arrangement*, WO2021162552, **2020**;
25. van Putten, R.; Uslamin, E.A.; Pidko, E.A., *Automated high-resolution sampling and multi-mode operando spectroscopy of (bio-)chemical reactions for kinetic analysis, reaction characterization, and quality control*, *Invention Disclosure* **2021**, *1*, 100002.
